# Comprehensive Analysis of ANLN in Human Tumors: A Prognostic Biomarker Associated with Cancer Immunity

**DOI:** 10.1155/2022/5322929

**Published:** 2022-03-17

**Authors:** Xiaoyu Zhang, Lin Li, Shaozhuo Huang, Weixin Liao, Jing Li, Zexuan Huang, Yuehua Huang, Yifan Lian

**Affiliations:** ^1^Department of Infectious Diseases, The Third Affiliated Hospital of Sun Yat-Sen University, Guangzhou, China; ^2^Guangdong Provincial Key Laboratory of Liver Disease Research, The Third Affiliated Hospital of Sun Yat-Sen University, Guangzhou, China; ^3^Department of General Surgery, The Third Affiliated Hospital of Sun Yat-Sen University, Guangzhou, China

## Abstract

**Background:**

Anillin (ANLN), a ubiquitously expressed actin-binding protein, plays a critical tumor-promoting role in cell growth, migration, and cytokinesis. Numerous studies have suggested that ANLN is upregulated in many cancer types, as well as significantly associated with patient prognosis and malignant cancer characteristics. Herein, we performed an integrated pan-cancer analysis of ANLN and highlighted its underlying mechanism, which may benefit further exploration of the potential therapeutic options for cancer.

**Methods:**

ANLN expression data were extracted from online databases, including TCGA, GTEx, and CCLE databases. The TIMER database was used to study the association between ANLN expression with immune checkpoint genes and immunocyte infiltration. The ScanNeo pipeline was adopted for neoantigen discovery. KEGG analysis and the STRING tool were used to elucidate the potential mechanism of ANLN in cancer development.

**Results:**

ANLN is abnormally overexpressed in almost all cancer tissues compared with normal tissues. The high-ANLN expression level was positively associated with various malignant characteristics, suggesting its potential role in the immune microenvironment and poor prognosis. In addition, ANLN expression was correlated with the number of neoantigens and different phosphorylation pattern in various cancer types, revealing a functional role of genetic mutation accumulation and high phosphorylation in ANLN-mediated oncogenesis. Moreover, we found that ANLN was an important regulatory factor participating in many signaling events, especially the cell cycle and nucleocytoplasmic transport pathways.

**Conclusions:**

ANLN expression is generally overexpressed in various types of cancers, and it may have an important influence on tumor progression and development. ANLN expression is significantly associated with the immune checkpoint biomarkers and tumor immunity. Together, these findings suggest that ANLN may be a predictive marker for patient prognosis across cancers.

## 1. Introduction

Due to the intricacy of tumorigenesis, an increasing number of studies have performed pan-cancer analyses of interesting genes and evaluated their relevance to the prognosis of survival and pathogenesis in cancer patients. Pan-cancer analysis was feasibly applied with the aid of many publicly available databases, such as TCGA (The Cancer Genome Atlas) and GEO (Gene Expression Omnibus), containing different datasets of gene function for diverse cancers [1–3].

Anillin (ANLN) is a highly evolutionarily conserved protein involved in a variety of cellular processes, involving in fitting cleavage furrows together during the entire division, especially cytokinesis [4, 5]. ANLN participates in the regulation of the anaphase and telophase of the cell cycle, by connecting with myosin, RhoA, actin, and septins to help the assembly of cleavage furrow and affecting cell polarity and motor ability [6–8]. Existing studies have shown that ANLN is necessary for tumor growth. Our previous study revealed that knockdown of ANLN leads to the suppression of cell proliferation, migration, and invasion of hepatocellular carcinoma [9]. ANLN is highly expressed in diverse human tissues and cells [10], and it is considered an early predictor for cancer diagnosis, such as bladder urothelial carcinoma [11], colorectal cancer [12], pancreatic ductal adenocarcinoma [13], non-small cell lung cancer [14], and breast cancer [15]. Although ANLN has been suggested to be a promising new target in cancer treatment, no pan-cancer analysis of this actin-binging protein has been reported. In the present study, we provided evidence of an association between ANLN expression and various tumor types based on a combined multidatabase. The mass of data has been provided by the unprecedented bioinformatics revolution, and it can be utilized to display the whole landscape containing a variety of genes and different tumor types [16]. Pan-cancer analysis has been aimed at analyzing the gene function and expression, prognosis of survival, and gene mutation of tumors in many databases [17].

In this study, we utilized multiple databases to explore the function of ANLN in various human tumors by evaluating the its RNA and protein expression patterns and the associations with prognosis of survival, tumor cell immune infiltration, immune neoantigens, and immune checkpoints. We also analyzed ANLN-related gene alterations, protein phosphorylation, and signaling pathway enrichment. Through this systematic analysis, an ANLN-dominated tumorigenic molecular mechanism and immune correlation were highlighted.

## 2. Materials and Methods

### 2.1. Sample Information and Pan-Cancer Expression Analysis of ANLN

The ANLN expression data in 31 normal tissues were evaluated in the Genotype-Tissue Expression (GTEx) program and obtained from the GTEx portal. The ANLN expression data in 21 tumor cell lines were investigated via the Cancer Cell Line Encyclopedia (CCLE) database (https://sites.broadinstitute.org/ccle/). We deployed the GTEx database (https://gtexportal.org/home/) and TCGA database (https://portal.gdc.cancer.gov/) to investigate the expression matrix of ANLN and the datasets of clinical information for tumor and normal tissues, respectively. The immune infiltrating cell score data of specific cancers were obtained from the Tumor IMmune Estimation Resource (TIMER) database (https://cistrome.shinyapps.io/timer/) to explore tumor immune infiltration analysis. The present study involved 33 cancer types which are listed in Table 1. All expression data passed log2 transformation. The UALCAN portal (http://ualcan.path.uab.edu/index.html), a website including interactive datasets used to obtain cancer-omics information, was used to explore a variety of proteomic datasets of the Clinical Proteomic Tumor Analysis Consortium (CPTAC) for performing clinical proteomics oncology [18]. By inputting “ANLN,” we investigated the total degree of proteomic expression and phosphor-protein expression of ANLN in cancer and normal tissues. Six available cancer datasets, including breast cancer, ovarian cancer, colon cancer, clear cell renal cell carcinoma, uterine body endometrial carcinoma, and lung adenocarcinoma, were utilized for clinical implication analysis.

### 2.2. Pan-Cancer Prognosis Analysis of ANLN Expression

We utilized the Kaplan-Meier (KM) (https://kmplot.com/analysis/) forest plots to show the association between ANLN expression and patient prognosis, including overall survival (OS), disease-free interval (DFI), and progression-free interval (PFI). Univariate survival analysis was used to estimate the logarithmic rank (log-rank) *P* value, hazard ratio (HR), and 95% confidence interval (CI).

### 2.3. ANLN-Related Immune Infiltration and Tumor Microenvironment (TME) Analysis

Purity-adjusted Spearman's test was performed to evaluated the associations between ANLN expression and immune cell infiltration of six cell types, including B cells, CD4^+^ T cells, CD8^+^ T cells, neutrophils, macrophages, and dendritic cells. The TIMER database was also used to visualize immune and genomics data, including infiltrating immune cell scores of 33 cancer types [19]. In addition, we assessed the following microenvironment-related scores, including the ESTIMATEScore, StromalScore, and ImmuneScore, for the respective patients' tumor samples. *P* value < 0.05 was considered statistically significant.

### 2.4. Correlation Analysis of ANLN in Immune Neoantigens and Immune Checkpoint Genes

Neoantigens are a group of molecules that are expressed on immune cells and are generated by genetic mutations, including gene fusions, deletion mutations, and point mutations. Immune checkpoints maintain their normal immune function to intervene in the level of immune activation *in vivo* [20]. We used ScanNeo to separately calculate the number of neoantigens on every tumor sample cell; subsequently, the association between ANLN expression and the number of antigens was presented. The relationship between ANLN expression levels and the degree of immune checkpoint markers in various cancers was analyzed through relevant modules. *P* < 0.05 and *R* > 0.20 were considered statistically significant.

### 2.5. Gene Enrichment Analysis of ANLN

First, we input “ANLN” in the protein name module and “Homo sapiens” in the organism module of the Search Tool for the Retrieval of Interacting Genes/Proteins (STRING) website (https://string-db.org/). Thereafter, we set the following primary parameters: the meaning of network edges (evidence), active interaction sources (experiments and database), minimum-required interaction score (low confidence (0.150)), and maximum number of interactors (no more than 50 interactors in the 1st shell). Finally, we obtained the available related proteins of ANLN.

We searched for the first 100 related ANLN-targeting genes across the “Similar Gene Detection” module of Gene Expression Profiling Interactive Analysis 2 (GEPIA2) (http://gepia2.cancer-pku.cn/), which contained TCGA tumor and normal tissue datasets. We then utilized the “correlation analysis” module of GEPIA2 to obtain selected genes and a pairwise Pearson correlation analysis of ANLN. We generated the dot plot through log2 TPM, and the *P* value and *R* value were calculated. In addition, we applied the “Gene_Corr” module of TIMER2 to generate the heat map data of the selected genes, including the partial correlation and *P* value in the purity-adjusted Spearman's rank correlation test. Jvenn, an interactive Venn diagram viewer [21], was utilized to conduct an intersection analysis to compare the relevant and connected genes of ANLN. The enriched pathways were determined via Kyoto Encyclopedia of Genes and Genomes (KEGG) and Gene Ontology (GO) analyses. The thresholds of analysis were ∣NES | >1, *P* value < 0.05, and FDR < 0.25. We certified pathways as substantially enriched if they satisfied the subconditions.

### 2.6. Statistical Analysis

The Kruskal-Wallis test was utilized to investigate ANLN expression levels in various tumor tissues and cancer cell lines. The difference of ANLN expression levels in tumor and normal tissues were assessed by the *t*-test. In survival analysis, the HR and *P* value were computed by the univariate Cox regression method. The relationship between ANLN expression and immunocyte infiltration level was analyzed via Spearman's correlation method. Pearson's correlation analysis was utilized to evaluate the relationship between the ANLN expression and immune infiltration scores, the number of tumor neoantigens, and immune checkpoint genes. The significance threshold of all statistical analyses was *P* < 0.05.

## 3. Results

### 3.1. Analysis of Differential Expression of ANLN in Pan-Cancer Tissues and Normal Tissues

**Figure 1:**
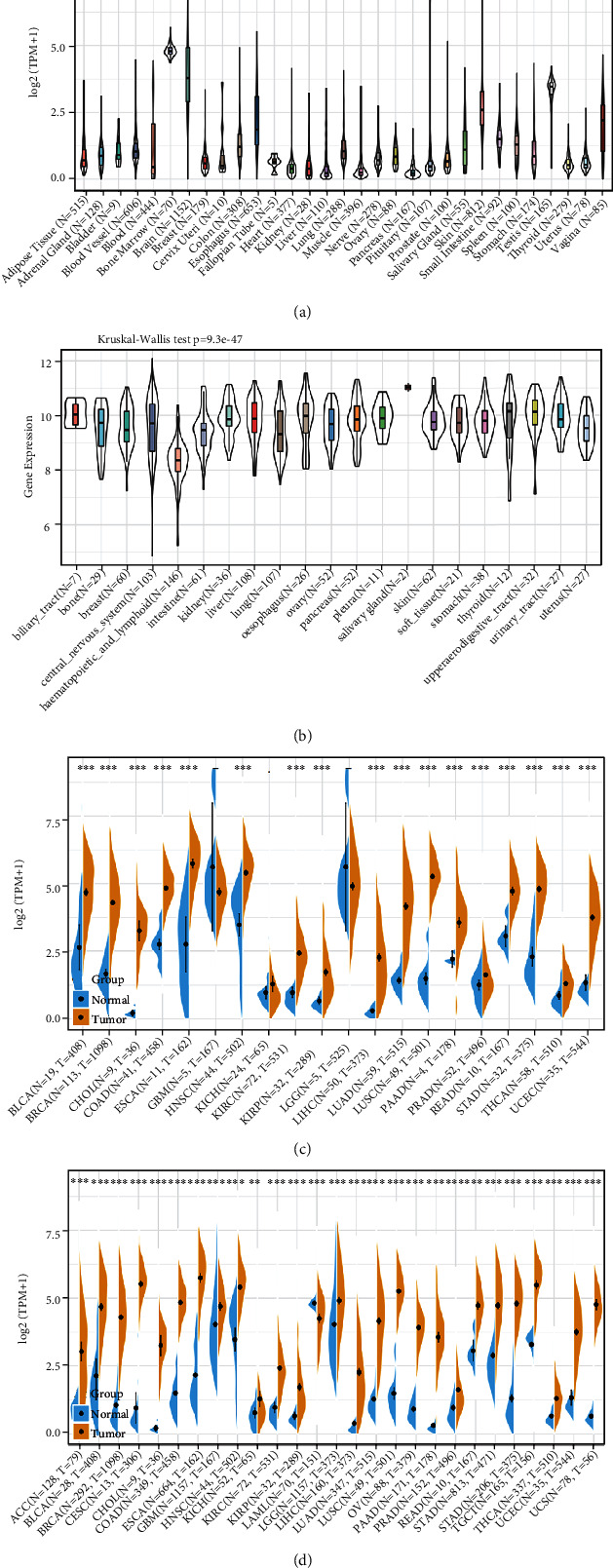
Analysis of differential expression of ANLN in pan-cancer tissues and normal tissues. ANLN mRNA expression level in (a) 31 types of normal tissues; (b) 21 kinds of cancer cell lines in CCLE database; (c) 20 types of cancer and normal tissues in TCGA database; (d) tumor and normal tissues in TCGA combined with GTEx database. ^∗∗^*P* < 0.01, ^∗∗∗^*P* < 0.001.

The present study performed a systematic analysis of the oncogenic influence of ANLN expression in human. As shown in Figure 1(a), we utilized the GTEx database to determine the expression pattern of ANLN in different tissues. We subsequently evaluated ANLN expression levels in 21 different human cancer cell lines from the CCLE database (Figure 1(b)). The results from TCGA database displayed the discrepancy of ANLN expression levels in tumor and adjacent normal tissues in individual cancer samples, thereby revealing ANLN expression specificity (Figure 1(c)). Due to the insufficient sample size of normal tissues in TCGA, we combined the data of normal and tumor tissues of GTEx and TCGA databases to evaluate the differential expression of ANLN in 27 cancer types (Figure 1(d)). The above analyses have shown that ANLN is abnormally overexpressed in almost all cancer tissues compared with normal tissues.

### 3.2. Pan-Cancer Analysis of the Prognostic Value of ANLN Expression

**Figure 2:**
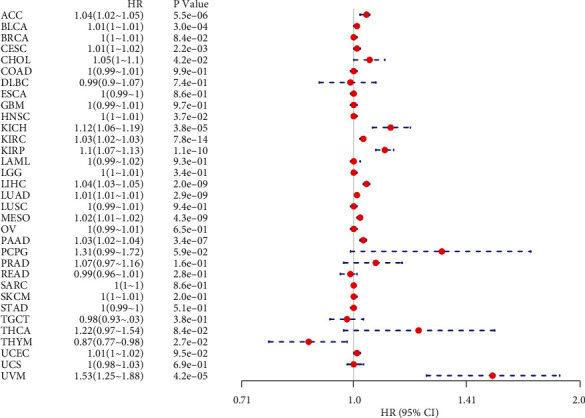
Pan-cancer analysis of the prognostic value of ANLN expression. A forest plot showing the HR and 95% CIs of ANLN expression associated with OS across cancers. Circles represent the HR, and the horizontal dotted lines extend from the lower limit to the upper limit of the 95% CI of the HR.

**Figure 3:**
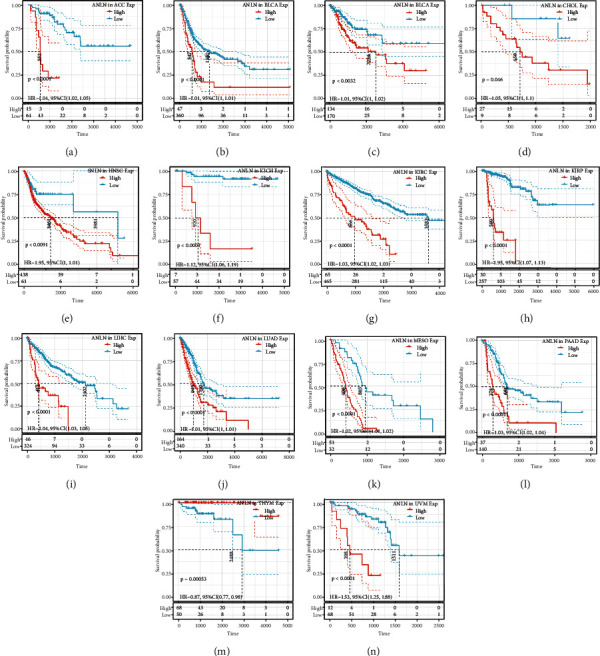
High ANLN expression was significantly associated with poor OS for most cancer patients. KM curves of patient OS split by high and low ANLN expressions within the following cancer types: (a) ACC, (b) BLCA, (c) CESC, (d) CHOL, (e) HNSC, (f) KICH, (g) KIRC, (h) KIRP, (i) LIHC, (j) LUAD, (k) MESO, (l) PAAD, (m) THYM, and (n) UVM.

We next analyzed the association of ANLN expression and cancer prognosis by utilizing one-way Cox regression analysis. The cancer cases were classified into two subgroups based on the ANLN expression levels. The forest plots across the 33 tumors demonstrated that ANLN expression had a significant impact on the OS of patients with ACC, BLCA, CESC, CHOL, KICH, KIRC, KIRP, LIHC, LUAD, MESO, PAAD, UVM, and THYM. The results also indicated that ANLN expression was mainly related to poor prognosis in most cancer types, except THYM (Figure 2). As shown in Figure 3, we further evaluated ANLN expression levels associated with the patient outcomes by utilizing the KM plotter portal and the log-rank method. The data indicated that high expression of ANLN was significantly associated with poor OS for patients with ACC (HR = 1.04, *P* < 0.001), BLCA (HR = 1.01, *P* < 0.001), CESC (HR = 1.01, *P* = 0.002), CHOL (HR = 1.05, *P* = 0.042), KICH (HR = 1.12, *P* < 0.001), KIRC (HR = 1.03, *P* < 0.001), KIRP (HR = 1.1, *P* < 0.001), LIHC (HR = 1.04, *P* < 0.001), LUAD (HR = 1.01, *P* < 0.001), MESO (HR = 1.02, *P* < 0.001), PAAD (HR = 1.03, *P* < 0.001), and UVM (HR = 1.53, *P* < 0.001). However, increased levels of ANLN indicated better patient outcomes in THYM (HR = 0.87, *P* = 0.027). Similarly, the association of ANLN expression with patient DFI was also investigated, demonstrating that ANLN expression influenced six tumor types, including PAAD, KICH, BRCA, KIPP, LIHC, and THCA (Figure S1A). In addition, KM curves also found that ANLN overexpression was associated with poor DFI in BRCA (HR = 1.01, *P* = 0.001), KIRC (HR = 1.11, *P* = 0.018), KIRP (HR = 1.09, *P* < 0.0001), LIHC (HR = 1.02, *P* < 0.0001), PAAD (HR = 1.04, *P* < 0.0001), and THCA (HR = 1.04, *P* < 0.0001) (Figure S1B-S1G). Moreover, a forest plot indicated that ANLN expression significantly affected patient PFI in many cancers, such as ACC, BLCA, BRCA, CESC, HNCS, KICH, KIRC, KIRP, LIHC, LUAD, MESO, PAAD, PCPG, PRAD, THCA, UCEC, and UVM (Figure S2A). Subsequently, KM curves confirmed that ANLN overexpression was associated with poor patient DFI in ACC (HR = 1.04, *P* = 1*e* − 04), BLCA (HR = 1.01, *P* < 0.0001), BRCA (HR = 1, *P* = 0.0014), CESC (HR = 1.01, *P* = 0.0072), HNCS (HR = 1, *P* = 0.0086), KICH (HR = 1.14, *P* < 0.0001), KIRC (HR = 1.02, *P* < 0.0001), KIRP (HR = 1.07, *P* < 0.0001), LIHC (HR = 1.02, *P* < 0.0001), LUAD (HR = 1.01, *P* = 0.00063), MESO (HR = 1.01, *P* < 0.0001), PAAD (HR = 1.03, *P* < 0.0001), PCPG (HR = 1.3, *P* < 0.0001), PRAD (HR = 1.06, *P* < 0.0001), THCA (HR = 1.37, *P* < 0.0001), UCEC (HR = 1.01, *P* = 0.0022), and UVM (HR = 1.56, *P* < 0.0001) (Figure S2B-S2R). The above data suggested that a higher expression level of ANLN predicted a poorer prognosis in different tumors, except for THYM. Overall, these data suggested that ANLN may be a prognostic biomarker associated with patient OS, DFI, and PFI in human cancers.

### 3.3. Pan-Cancer Analysis of ANLN Protein Phosphorylation

**Figure 4:**
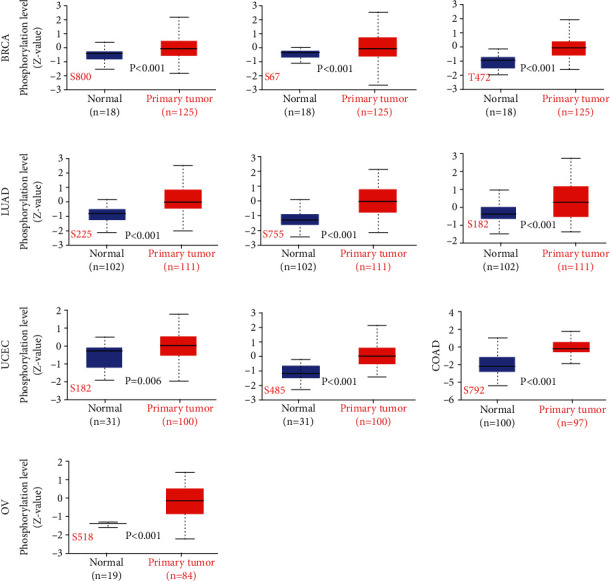
Tumor-associated protein phosphorylation of ANLN. Box plot representation of ANLN phosphorylation levels at different amino acid residues in BRCA, LUAD, UCEC, COAD, and OV as indicated.

The phosphorylation-dephosphorylation cascade is regarded as an essential condition in oncogenesis. Several types of ANLN protein phosphorylation, including phosphorylation of serine and threonine residues, were identified via the CPTAC dataset. We subsequently compared the different phosphorylation features of ANLN in normal and tumor tissues by utilizing the CPTAC dataset. The ANLN phosphorylation data in various tumor types were shown in Figure 4. The ANLN phosphorylation level was significantly increased in various tumors. The following residues had higher phosphorylation levels: S182 and S485 in UCEC; S182, S255, and S755 in LUAD; S67, T472, and S800 in BRCA; S518 in OV; and S792 in COAD (Figure 4).

### 3.4. Pan-Cancer Association Analysis of ANLN Expression and Tumor Immune Infiltration

**Figure 5:**
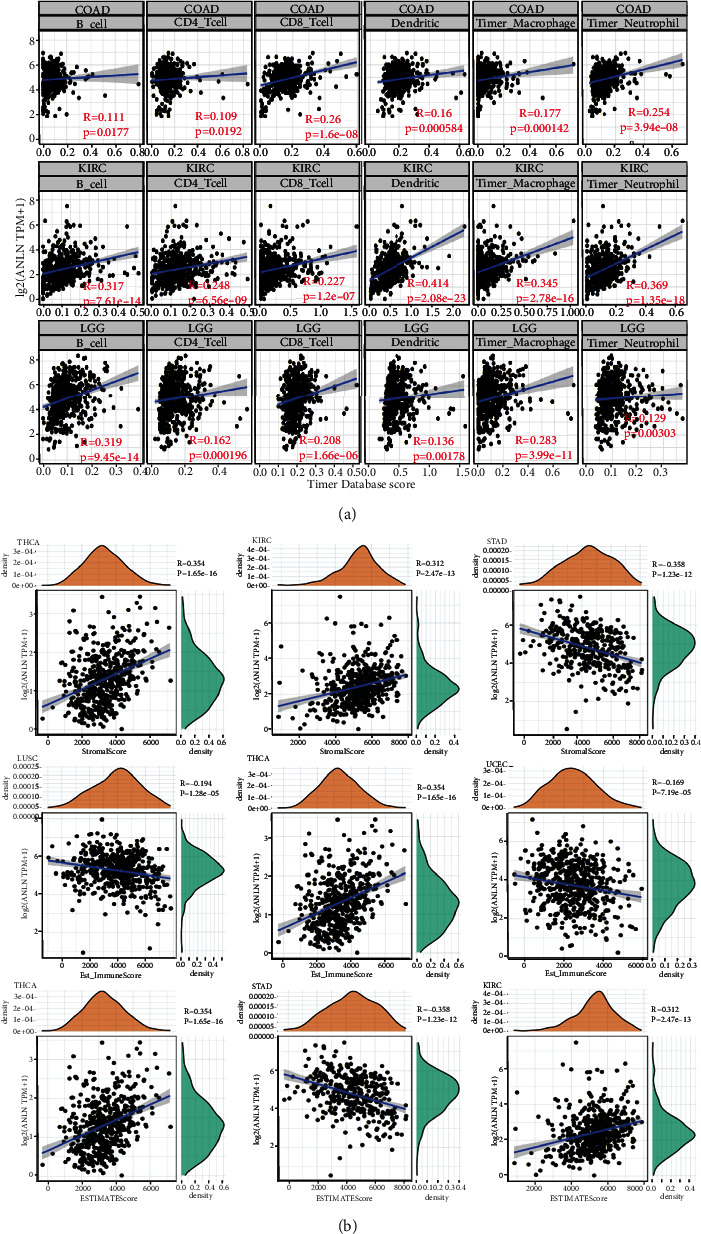
Pan-cancer association analysis of ANLN expression and tumor immune infiltration. (a) Dot plots show the correlation of ANLN expression and the infiltration of six types of immunocytes in COAD (a1), KIRC (a2), and LGG (a3). (b) Correlation of ANLN expression with StromalScore (b1), ImmuneScore (b2), and ESTIMATEScore (b3) in the top 3 cancer types.

The degree of tumor immune infiltration of different immune cells is significantly associated with cancer initiation, progression, and metastasis [22]. TME composed of the external matrix, various cells, and relevant factors play an important role across cancers and affect the diagnosis, clinical treatment sensitivity, and survival of cancer patients. Therefore, we explored the association of ANLN expression with the infiltration level of immune cells in various tumors, particularly in COAD, LGG, and KIRC. We found that the ANLN expression was closely correlated with CD8^+^ T cells (*R* = 0.26, *P* < 0.001), macrophages (*R* = 0.177, *P* < 0.001), neutrophils (*R* = 0.254, *P* < 0.001), and dendritic cells (*R* = 0.16, *P* < 0.001) in COAD (Figure 5(a)). ANLN expression was also positively associated with immune cells, including CD4^+^ T cells (*R* = 0.248, *P* < 0.001), CD8^+^ T cells (*R* = 0.227, *P* < 0.001), macrophages (*R* = 0.345, *P* < 0.001), B cells (*R* = 0.317, *P* < 0.001), neutrophils (*R* = 0.369, *P* < 0.001), and dendritic cells (*R* = 0.414, *P* < 0.001) in KIRC (Figure 5(a)). As shown in Figure 5(a), ANLN expression was significantly associated with CD4^+^ T cells (*R* = 0.162, *P* < 0.001), CD8^+^ T cells (*R* = 0.208, *P* < 0.001), B cells (*R* = 0.319, *P* < 0.001), macrophages (*R* = 0.238, *P* < 0.001), and dendritic cells (*R* = 0.136, *P* < 0.001) in LGG. Additionally, the association of ANLN expression with various developmental processes in TME was analyzed. We used the estimate package in R to calculate the StromalScore and ImmuneScore in tumor samples.  Figure 5(b) showed the first three cancers, namely, THCA (*R* = 0.354, *P* < 0.001), KIRC (*R* = 0.312, *P* < 0.001), and STAD (*R* = −0.358, *P* < 0.001), in which ANLN expression was most closely associated with the ImmuneScore. The first three cancers with ANLN expression closely linked to StromalScore were LUSC (*R* = −0.194, *P* < 0.001), THCA (*R* = 0.354, *P* < 0.001), and UCEC (*R* = −0.169, *P* < 0.001). Thus, ANLN expression was negatively related to the ImmuneScore in STAD but positively related to the ImmuneScore in THCA and KIRC. In addition, the StromalScores of LUSC and UCEC were negatively associated with ANLN expression, but in THCA, it showed a positive association.

### 3.5. Correlation Analysis of ANLN Expression with Immune Neoantigens and Immune Checkpoints across Cancers

**Figure 6:**
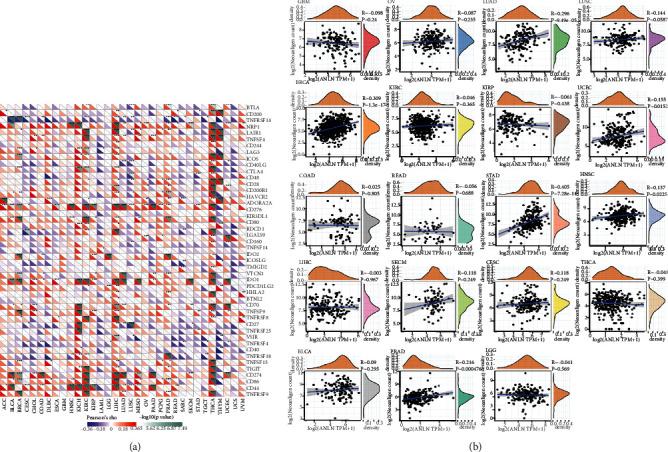
Pan-cancer correlation analysis of ANLN expression with immune characteristics. (a) The correlation matrix shows the relationships between ANLN expression and immune checkpoint genes across cancers. Each cell in the matrix represents a correlation between ANLN expression with an immune checkpoint molecule (the list on the right side of the matrix) in a certain type of cancer (the list at the bottom of the matrix). The correlation coefficient and *P* value are shown in the cell by indicated color. ^∗^*P* < 0.05, ^∗∗^*P* < 0.01, ^∗∗∗^*P* < 0.001. (b) Correlation of ANLN expression with the number of neoantigens across different types of cancers.

A series of molecules expressed in immune cells are regarded as immune checkpoints that are essential in regulating autoimmunity and can mediate the level of immune activation [23]. In tumor cells, mutated genes encode nascent antigens, which are considered cancer neoantigens. Exploiting the immune activity of tumor neoantigens promotes the synthesis of neoantigen vaccines [24]. As shown in Figure 6(a), the associations between ANLN expression and over 40 immune checkpoint genes were explored in various tumor types. The results suggested that ANLN expression was strongly correlated with immune checkpoints, especially in LIHC, THCA, and KIRC. This indicated that in some cancers, ANLN expression had an important role in tumor immunity by regulating the expression pattern of immune checkpoints. In addition, after calculating the quantity of neoantigens of every cancer type, we demonstrated that ANLN expression was positively associated with the quantity of neoantigens in LUAD (*R* = 0.144, *P* < 0.01), BRCA (*R* = 0.309, *P* < 0.01), STAD (*R* = 0.405, *P* < 0.01), UCEC (*R* = 0.155, *P* = 0.0152), and HNSC (*R* = 0.137, *P* = 0.0225) (Figure 6(b)).

### 3.6. Enrichment Analysis of ANLN-Related Partners

**Figure 7:**
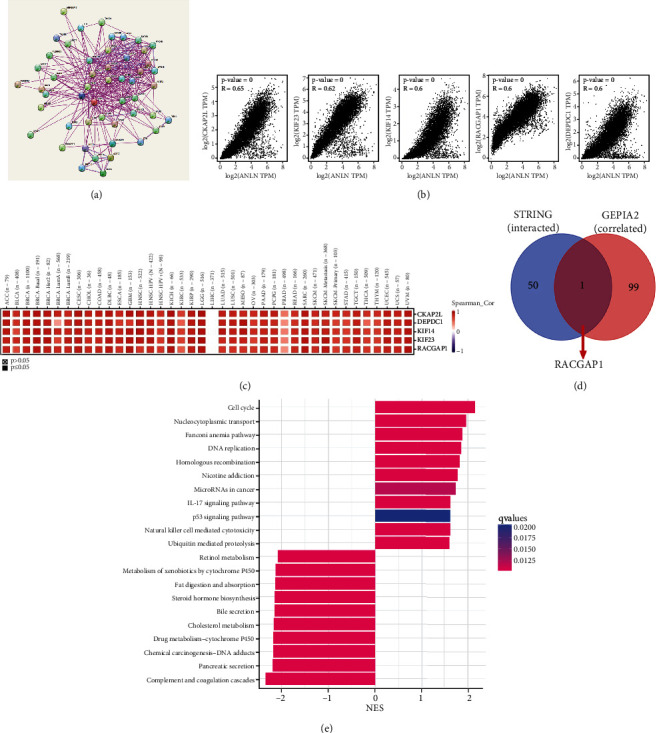
Analysis of ANLN-related genes and pathway enrichment. (a) STRING protein network map of top 50 experimentally determined ANLN-binding proteins. Each colored nodes indicate one individual protein identified. (b) Expression correlation between ANLN and the representative genes (CKAP2L, KIF23, KIF14, RACGAP1, and DEPDC1) of the top ANLN-correlated genes in the TCGA database generated by the GEPIA2 tool. (c) Heatmap representation of the expression correlation between ANLN and CKAP2L, KIF23, KIF14, RACGAP1, and DEPDC1 in the individual cancer type of all TCGA tumors. (d) An intersection analysis of the ANLN-interacted (by STRING database) and ANLN-correlated (by GEPIA2) genes. RACGAP1 is the only intersected genes of the above two datasets. (e) KEGG pathway analysis based on the ANLN-interacted and ANLN-correlated genes.

We next investigated ANLN-interacting proteins and the genes associated with ANLN expression for a series of pathway enrichment analyses to observe the molecular mechanism of ANLN in the tumor progression. Using the STRING tool, we obtained 50 proteins that interacted with ANLN and the relevant protein network was displayed in Figure 7(a). We analyzed the first 100 genes that correlated with ANLN expression in cancer expression datasets of TCGA by using the GEPIA2 tool. We showed that ANLN expression was positively associated with the DEPDC1 (*R* = 0.6), KIF14 (*R* = 0.6), KIF23 (*R* = 0.62), RACGAP1 (*R* = 0.6), and CKAP2L (*R* = 0.65) genes (all *P* < 0.001) (Figure 7(b)). In addition, ANLN was closely associated with the above five genes in various tumor types, as shown in the corresponding heat map (Figure 7(c)). A Venn diagram showed that RACGAP was the intersection gene of the above two datasets (Figure 7(d)). Finally, we classified the tumor samples into subgroups according to different ANLN expression levels to observe its influence on cancers. We identified the enriched biological processes and signaling pathways in these two groups by KEGG analysis (Figure 7(e)). We generated the visual pathway map based on the permutation of the NES score. The pathway map showed that the cell cycle, nucleocytoplasmic transport, and Fanconi anaemia pathway were positively related to ANLN expression. In contrast, complement and coagulation cascades, pancreatic secretion, and chemical carcinogenesis-DNA adducts were significantly negatively related to ANLN expression (Figure 7(e)).

## 4. Discussion

In recent years, cancer has become a more prominent public health problem. The number of patients with cancers in the world has exceeded 90 million [25]. To better understand the underlying molecular mechanisms of cancer and to devise better treatments, many studies have performed pan-cancer analysis to detect new prognostic and diagnostic biomarkers. Previous studies had focused on the association between ANLN expression and the malignant progression of tumors, demonstrating that ANLN is upregulated in various cancers. Herein, we first presented a systematic and comprehensive analysis of ANLN in human carcinoma. We analyzed ANLN expression in 33 different cancers using public databases including TCGA, CPTAC, and GEO. We then summarized the gene expression, protein phosphorylation, and enrichment analysis of ANLN-related partners as well as the association between ANLN expression levels with immune cell infiltration, immune neoantigens, and immune checkpoint markers.

ANLN is a unique scaffolding protein that has been demonstrated to be an important factor in cell division, and it has a close relationship with major cytoskeletal structures. ANLN is usually degraded after mitosis and remains in the nucleus during mitosis. Overexpression of ANLN may disrupt the normal regulatory mechanism by influencing the actin-myosin cytoskeleton in events other than cytokinesis, thus directly promoting cancer progression [5]. Numerous studies have implied that ANLN is overexpressed in a variety of human cancers. By analyzing ANLN expression levels using the GTEx portal, CCLE database, and TCGA database, we confirmed that ANLN was overexpressed in various cancers compared with adjacent normal tissues and we also showed that ANLN played an important role as a prognostic marker of various cancers, agreeing with those previous reports on lung cancer [26], pancreatic cancer [13], and breast cancer [15]. However, the upstream factors regulating the expression of ANLN and why ANLN is consistently upregulated in various cancer types are undefined and await further investigations.

ANLN phosphorylation status plays a vital role in cytokinesis. Previous studies have shown that ANLN is an essential cytoskeletal protein that combines the actomyosin ring with the membrane and its organizer, RhoA [5, 8]. ANLN is highly phosphorylated during mitosis [27]. Furthermore, ANLN phosphorylation is positively involved in its cellular distribution and function during cytokinesis, such as meiotic division [28, 29]. In the present study, we investigated several types of ANLN protein phosphorylation and compared the phosphorylation levels of ANLN between the main tumor and normal tissues using the CPTAC database. Our study showed that the phosphorylation level of ANLN was higher in some cancers, such as ovarian cancer, lung cancer, and breast cancer. It has been reported that phosphorylation regulates the interaction between ANLN and the equatorial membrane, allowing ANLN to recruit Rho and its regulators upstream and downstream in time to ensure that the cells divide successfully [27]. Further investigations are required to determine the molecular mechanisms of the protein phosphorylation level of ANLN in tumors.

In recent years, scientists have focused on the TME. Immunity in tumorigenesis has an important influence on cancer therapy. The immune microenvironment, which is considered the “seventh marker feature” of cancer [30], includes T cells, dendritic cells, B cells, macrophages, and neutrophils. However, in the incipient stage of tumors, the TME, known as the inflammatory tumor microenvironment made up of tumor cells, inflammatory cells, and the stroma around the tumor, has a vital influence on tumor progression, angiogenesis, and genomic instability [31]. Consequently, we explored the roles that ANLN plays in the TME in human tumors. Our research revealed that the ANLN expression level was strongly associated with six infiltrating immune cells, including CD4^+^ T cells, dendritic cells, B cells, macrophages, CD8^+^ T cells, and neutrophils, in KIRC, COAD, and LGG. We further assessed the correlation between the expression levels of ANLN and TME in various cancers and found that it was negatively associated with the ImmuneScore in STAD but positively associated with the ImmuneScore in THCA and KIRC. The StromalScores in LUSC and UCEC were negatively correlated with ANLN expression levels, but the StromalScores in THCA were positively correlated with ANLN expression levels. These results were consistent with those of previous studies. For instance, ANLN depletion leads to familial acute respiratory distress syndrome (ARDS) [32]. In addition, IFN-*γ* induces upregulation of ANLN transcription in human epithelial cells [33]. Together, these results suggested that ANLN interacts with immune cells and this correlation warrants further in-depth studies.

At present, little evidence of ANLN expression associated with immunity is provided in cancers. In the present study, we investigated the various roles of ANLN in modulating tumor immunology in some tumors. The expression levels of ANLN correlated with more than 40 checkpoints in various tumors, and they were positively associated with LIHC, THCA, and KIRC. By analyzing the relationship between ANLN expression and the quantity of neoantigens, we observed that ANLN expression was positively correlated with LUAD, BRCA, STAD, UCEC, and HNSC, suggesting that ANLN may enhance immunity in some tumor types. Recent findings have shown that the role of ANLN is significant in the immune reaction via association between ANLN and KDR, which may serve as a potential predictor of breast cancer survival [34].

To analyze the molecular mechanism of the ANLN in the tumor progression, we further explored the function of ANLN in cancers by analyzing the enrichment of ANLN-relevant genes and proteins. Indeed, published works by others have reported expressional dysregulation of many signaling pathways after depletion of ANLN expression in breast, pancreatic, and bladder cancer cell lines [11, 13, 35]. For example, molecular pathways related to epithelial and epidermal cell differentiation are activated after ANLN knockout in breast cancer cells [35]; ECM adhesion and actin cytoskeleton pathways are dysregulated in pancreatic cells, which is consistent with the decreased cell motility following ANLN depletion in these cells [13]; changes of other noncanonical pathways like Toll-like receptor and cytokine signaling are also observed in bladder cancer cells [11]. Different from the abovementioned studies, our analyses showed that the cell cycle, nucleocytoplasmic transport, and Fanconi anaemia pathways were significantly positively correlated with ANLN expression. In addition, ANLN expression was strongly associated with that of DEPDC, KIF14, KIF23, RACGAP1, and CKAP2L genes across cancers. Previous reports indicated that ANLN could promote tumor progression through its well-studied function as a scaffold protein in regulating cytokinesis. However, recent studies have provided novel evidence that ANLN may serve as a nuclear regulator for transcriptional reprogramming and control intercellular adhesion and cellular motility to alter the phenotypic plasticity of cancer cells, thus exerting its tumor-promoting functions [36, 37]. In line with the above description, our result also demonstrated that RACGAP1 was a highly relevant gene to ANLN expression across cancers by the intersection analysis of STRING and GEPIA2 datasets. RACGAP1 has been identified as a prominent activator of tumor invasion and metastasis in various cancers, including uterine carcinosarcoma [38], breast cancer [39], and ovarian cancer [40]. It could be speculated that ANLN induces the motility of cancer cells through regulating RACGAP1 expression in an undefined manner. Similar to the novel regulator function of ANLN in cell motility, recent studies also revealed the emerging role of ANLN in regulating cancer stemness, a phenotypic hallmark which shared many characteristics with cellular metastasis. In breast cancer, ANLN upregulation markedly enhances the self-renewal potential of epithelial-type MCF10AneoT cells, whereas loss of ANLN decreases stem/progenitor properties of mesenchymal-type MDA-MB-231 and BT549 breast cancer cells [35]. In addition, ANLN was used as a biomarker to monitor the late dividing retinal precursors and stem cells when fused with the eGFP reporter in an *in vivo* zebrafish model [41]. Though the underlying mechanism remains unknown, ANLN-regulating cancer stemness could be associated with expressional regulation of a stem cell-specific transcriptional network [35, 42]. Since ANLN expression was highly correlated with the nucleocytoplasmic transport pathway, the activity of many transcriptional regulators related to stemness thus could possibly be altered under ANLN differential expression. However, further investigations are needed to verify the hypothesis.

## 5. Conclusion

In conclusion, the present study demonstrated that ANLN expression is closely related to poor prognosis in various cancers. The high ANLN expression level and elevated phosphorylation of ANLN may be potential factors for tumor progression. ANLN expression is significantly associated with the immune checkpoint biomarkers and tumor immunity. Together, these findings suggest that ANLN may be a predictive marker for patient prognosis across cancers.

## Figures and Tables

**Table 1:** List of abbreviations of cancer names used in this study.

Cancer abbreviations	Corresponding meanings of caner abbreviations
ACC	Adrenocortical carcinoma
BLCA	Bladder urothelial carcinoma
BRCA	Breast invasive carcinoma
CESC	Cervical squamous cell carcinoma and endocervical adeno carcinoma
CHOL	Cholangiocarcinoma
COAD	Colon adenocarcinoma
READ	Rectum adenocarcinoma esophageal carcinoma
DLBC	Lymphoid neoplasm diffuse large B-cell lymphoma
ESCA	Esophageal carcinoma
GBM	Glioblastoma multiforme
HNSC	Head and neck squamous cell carcinoma
KICH	Kidney chromophobe
KIRC	Kidney renal clear cell carcinoma
KIRP	Kidney renal papillary cell carcinoma
LAML	Acute myeloid leukemia
LGG	Brain lower-grade glioma
LIHC	Liver hepatocellular carcinoma
LUAD	Lung adenocarcinoma
LUSC	Lung squamous cell carcinoma
MESO	Mesothelioma
OV	Ovarian serous cystadenocarcinoma
PAAD	Pancreatic adenocarcinoma
PCPG	Pheochromocytoma and paraganglioma
PRAD	Prostate adenocarcinoma
READ	Rectum adenocarcinoma
SARC	Sarcoma
SKCM	Skin cutaneous melanoma
STAD	Stomach adenocarcinoma
STES	Stomach and esophageal carcinoma
TGCT	Testicular germ cell tumors
THCA	Thyroid carcinoma
THYM	Thymoma
UCEC	Uterine corpus endometrial carcinoma
UVM	Uveal melanoma

## Data Availability

The data used in this study and analyzed were obtained from the publicly available databases.

## Abbreviations

ANLN: Anillin
CCLE: Cancer Cell Line Encyclopedia
CI: Confidence interval
CPTAC: Clinical Proteomic Tumor Analysis Consortium
DFI: Disease-free interval
GEO: Gene Expression Omnibus
GEPIA2: Gene Expression Profiling Interactive Analysis 2
GO: Gene Ontology
GTEx: Genotype-Tissue Expression
HR: Hazard ratio
KEGG: Kyoto Encyclopedia of Genes and Genomes
KM: Kaplan-Meier
log-rank: Logarithmic rank
OS: Overall survival
PFI: Progression-free interval
STRING: Search Tool for the Retrieval of Interacting Genes/Proteins
TCGA: The Cancer Genome Atlas
TIMER: Tumor IMmune Estimation Resource
TME: Tumor microenvironment.

## Supplementary Materials

Supplementary Materials Figure S1: pan-cancer association analysis of ANLN expression with patient DFI. (A) A forest plot showing the HR and 95% CIs of ANLN expression associated with DFI across cancers. Circles represent the HR and the horizontal dotted lines extend from the lower limit to the upper limit of the 95% CI of the HR. (B–G) KM curves of patient DFI split by high and low ANLN expressions within the following cancer types: (b) BRCA, (c) KIRC, (d) KIRP, (e) LIHC, (f) PAAD, and (g) THCA. Figure S2: pan-cancer association analysis of ANLN expression with patient PFI. (A) A forest plot showing the HR and 95% CIs of ANLN expression associated with PFI across cancers. Circles represent the HR and the horizontal dotted lines extend from the lower limit to the upper limit of the 95% CI of the HR. (B–R) KM curves of patient PFI split by high and low ANLN expressions within the following cancer types: (b) ACC, (c) BLCA, (d) BRCA, (e) CESC, (f) HNSC, (g) KICH, (h) KIRC, (i) KIRP, (j) LIHC, (k) LUAD, (l) MESO, (m) PAAD, (n) PCPG, (o) PRAD, (p) THCA, (q) UCEC, and (r) UVM.
