# Supplementary figures and images for: Comprehensive Analysis of ANLN in Human Tumors: A Prognostic Biomarker Associated with Cancer Immunity
Source: Oxid Med Cell Longev. 2022 Mar 17;2022:5322929. doi: 10.1155/2022/5322929 (PMC8947880; doi:10.1155/2022/5322929)

# Figure S1

## A

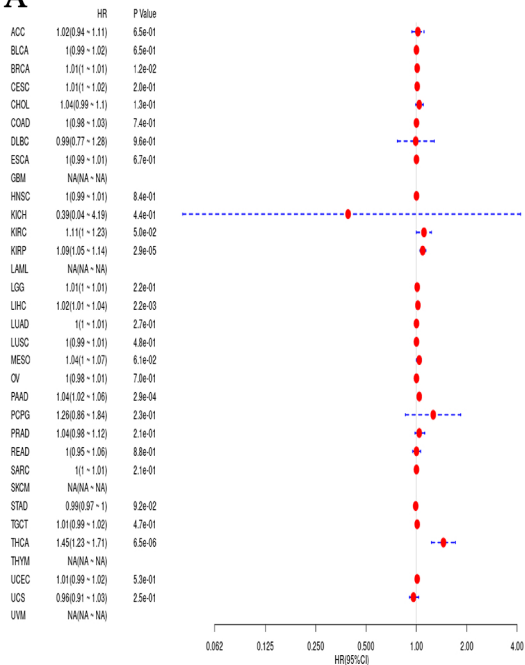

## B

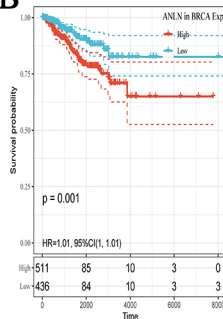

## C

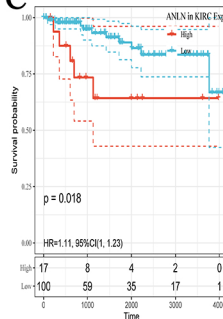

## D

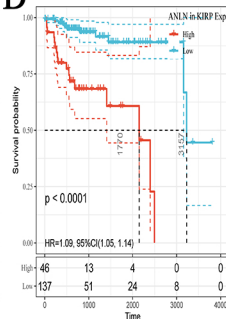

## E

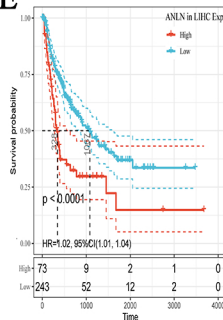

## F

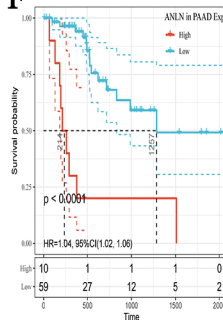

## G

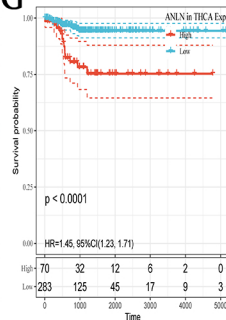

Supplement: Supplementary Materials — Figure S1: pan-cancer association analysis of ANLN expression with patient DFI. (A) A forest plot showing the HR and 95% CIs of ANLN expression associated with DFI across cancers. Circles represent the HR and the horizontal dotted lines extend from the lower limit to the upper limit of the 95% CI of the HR. (B–G) KM curves of patient DFI split by high and low ANLN expressions within the following cancer types: (b) BRCA, (c) KIRC, (d) KIRP, (e) LIHC, (f) PAAD, and (g) THCA. Figure S2: pan-cancer association analysis of ANLN expression with patient PFI. (A) A forest plot showing the HR and 95% CIs of ANLN expression associated with PFI across cancers. Circles represent the HR and the horizontal dotted lines extend from the lower limit to the upper limit of the 95% CI of the HR. (B–R) KM curves of patient PFI split by high and low ANLN expressions within the following cancer types: (b) ACC, (c) BLCA, (d) BRCA, (e) CESC, (f) HNSC, (g) KICH, (h) KIRC, (i) KIRP, (j) LIHC, (k) LUAD, (l) MESO, (m) PAAD, (n) PCPG, (o) PRAD, (p) THCA, (q) UCEC, and (r) UVM. [file 5322929.f1.zip › 5322929.f1.pdf]

Figure S2

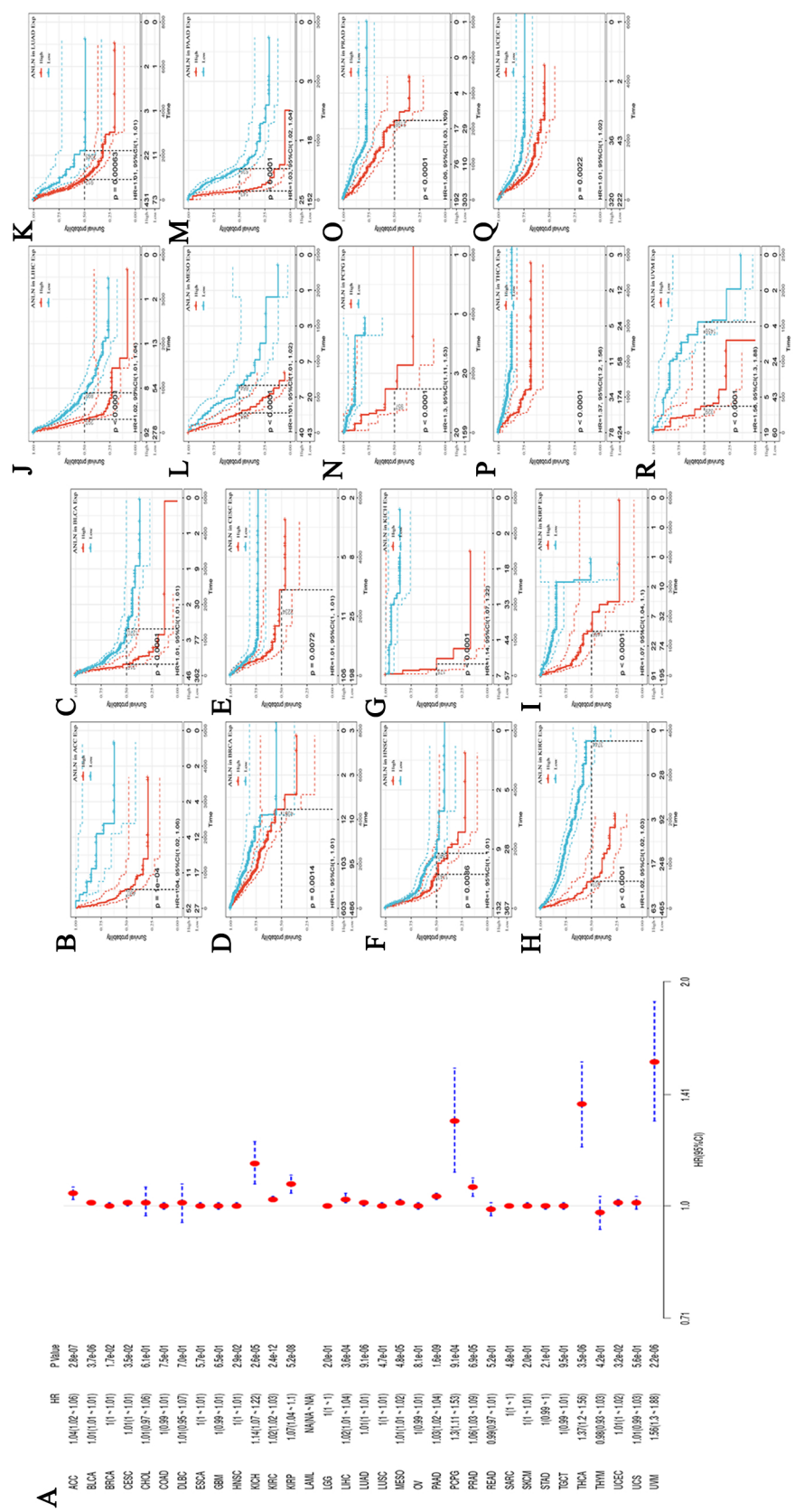

Supplement: Supplementary Materials — Figure S1: pan-cancer association analysis of ANLN expression with patient DFI. (A) A forest plot showing the HR and 95% CIs of ANLN expression associated with DFI across cancers. Circles represent the HR and the horizontal dotted lines extend from the lower limit to the upper limit of the 95% CI of the HR. (B–G) KM curves of patient DFI split by high and low ANLN expressions within the following cancer types: (b) BRCA, (c) KIRC, (d) KIRP, (e) LIHC, (f) PAAD, and (g) THCA. Figure S2: pan-cancer association analysis of ANLN expression with patient PFI. (A) A forest plot showing the HR and 95% CIs of ANLN expression associated with PFI across cancers. Circles represent the HR and the horizontal dotted lines extend from the lower limit to the upper limit of the 95% CI of the HR. (B–R) KM curves of patient PFI split by high and low ANLN expressions within the following cancer types: (b) ACC, (c) BLCA, (d) BRCA, (e) CESC, (f) HNSC, (g) KICH, (h) KIRC, (i) KIRP, (j) LIHC, (k) LUAD, (l) MESO, (m) PAAD, (n) PCPG, (o) PRAD, (p) THCA, (q) UCEC, and (r) UVM. [file 5322929.f1.zip › 5322929.f2.pdf]
